# Pioglitazone-Mediated Peroxisome Proliferator-Activated Receptor γ Activation Aggravates Murine Immune-Mediated Hepatitis

**DOI:** 10.3390/ijms21072523

**Published:** 2020-04-05

**Authors:** Rike Schulte, Dirk Wohlleber, Ludmilla Unrau, Bernd Geers, Christina Metzger, Annette Erhardt, Gisa Tiegs, Nico van Rooijen, Lukas C. Heukamp, Luisa Klotz, Percy A. Knolle, Linda Diehl

**Affiliations:** 1 Institute for Molecular Medicine and Experimental Immunology, University Hospital Bonn, Bonn, Germany; rike.schulte@gmail.com (R.S.); ga59siw@mytum.de (D.W.); c_metzger@gmx.net (C.M.); luisa.klotz@ukmuenster.de (L.K.); percy.knolle@tum.de (P.A.K.); 2 Institute of Molecular Immunology and Experimental Oncology, Technical University Munich, 81675, Munich, Germany; 3 Institute of Experimental Immunology and Hepatology, University Medical Center Hamburg-Eppendorf, 20246 Hamburg, Germany; l.unrau@uke.de (L.U.); b.geers@uke.de (B.G); annette.erhardt.ae@googlemail.com (A.E.); g.tiegs@uke.de (G.T.); 4 Department of Molecular Cell Biology, VU University Medical Center, 1081 HV Amsterdam, The Netherlands; nvanrooijen@clodronateliposomes.org; 5 Institute for Hematopathology Hamburg, 22547 Hamburg, Germany; heukamp@hp-hamburg.de; 6 Department of Neurology, University Hospital Münster, 48149 Münster, Germany

**Keywords:** TNFα, Kupffer cell, PPARγ, LPS, inflammation, Pioglitazone

## Abstract

The nuclear receptor peroxisome proliferator-activated receptor gamma (PPARγ) regulates target gene expression upon ligand binding. Apart from its effects on metabolism, PPARγ activity can inhibit the production of pro-inflammatory cytokines by several immune cells, including dendritic cells and macrophages. In chronic inflammatory disease models, PPARγ activation delays the onset and ameliorates disease severity. Here, we investigated the effect of PPARγ activation by the agonist Pioglitazone on the function of hepatic immune cells and its effect in a murine model of immune-mediated hepatitis. Cytokine production by both liver sinusoidal endothelial cells (IL-6) and in T cells ex vivo (IFNγ) was decreased in cells from Pioglitazone-treated mice. However, PPARγ activation did not decrease pro-inflammatory tumor necrosis factor alpha TNFα production by Kupffer cells after Toll-like receptor (TLR) stimulation ex vivo. Most interestingly, although PPARγ activation was shown to ameliorate chronic inflammatory diseases, it did not improve hepatic injury in a model of immune-mediated hepatitis. In contrast, Pioglitazone-induced PPARγ activation exacerbated D-galactosamine (GalN)/lipopolysaccharide (LPS) hepatitis associated with an increased production of TNFα by Kupffer cells and increased sensitivity of hepatocytes towards TNFα after in vivo Pioglitazone administration. These results unravel liver-specific effects of Pioglitazone that fail to attenuate liver inflammation but rather exacerbate liver injury in an experimental hepatitis model.

## 1. Introduction

The transcription factor nuclear receptor peroxisome proliferator-activated receptor gamma (PPARγ) has been shown to mediate anti-inflammatory effects on several immune cell types [1,2]. PPARγ heterodimerizes with the retinoid X receptor upon ligand binding and then binds to PPAR response elements in the promoter region of target genes. Additionally, PPARγ negatively affects pro-inflammatory cell signaling, either via competition for co-activators or via transrepression through physical interaction with pro-inflammatory transcription factors, in particular NF-κB [2,3]. PPARγ agonists can be activated by endogenous ligands, such as polyunsaturated fatty acids and prostanoids like the prostaglandin D2 (PG-D_2)_ metabolite 15-deoxy-D PG-J_2_ [4]. Additionally, several synthetic ligands exist, such as the anti-diabetic thiazolidinediones, e.g., Pioglitazone and Rosiglitazone, which can be used for the correction of metabolic disturbances in type II diabetes [5].

Various cell types of the immune system are affected in their function after PPARγ activation. In dendritic cells (DC), for instance, it prevents pro-inflammatory cytokine production and maturation, which can impede DC priming of CD4 T cells [6], and it affects DC migration and adhesion [7]. Also, in peripheral blood monocytes and peritoneal macrophages, PPARγ can downregulate the production of pro-inflammatory cytokines, like interleukin (IL) 1β, IL-6 and TNFα, and additionally affects the uptake and destruction of pathogens [8]. In T cells, PPARγ activation results in reduced proliferation and IL-2 production [4]. Furthermore, we could show that PPARγ activation interferes with retinoic acid receptor-related orphan receptor (ROR)γt transcription and thus, Th17 differentiation [9].

Liver disease is a major cause of morbidity and mortality worldwide. Both chronic and acute hepatitis can cause liver injury, which can lead to the development of liver cirrhosis and cancer. Hepatitis is largely mediated by immune effector mechanisms. PPARγ activation can have beneficial effects on disease onset and severity in various inflammatory disease models in the central nervous system, the gastrointestinal tract and the skin [9,10,11]. Thus, different hepatic immune cells that are implicated in the development of immune hepatitis may be potentially susceptible to the anti-inflammatory properties of PPARγ. Moreover, it has been reported that reduced liver injury after flavonoid treatment in the model of GalN/LPS hepatitis was associated with increased expression of PPARγ [12]. This prompted us to investigate whether pharmacological activation of PPARγ can augment the anti-inflammatory function of different hepatic immune cells and whether this influences liver injury in the murine model of immune-mediated GalN/LPS-induced hepatitis.

## 2. Results

### 2.1. PPARγ Activation Regulates Pro-Inflammatory Activity in liver sinusoidal endothelial cells (LSEC) and T Cells but not Kupffer Cells

PPARγ has been shown to have anti-inflammatory effects on different cells of the immune system. We have previously observed that PPARγ activation in DC leads to a diminished production of pro-inflammatory cytokines after TLR stimulation [6]. PPARγ activation in peritoneal macrophages also inhibits their immune function, resulting in reduced respiratory burst and TNFα production [2].

**Figure 1:**
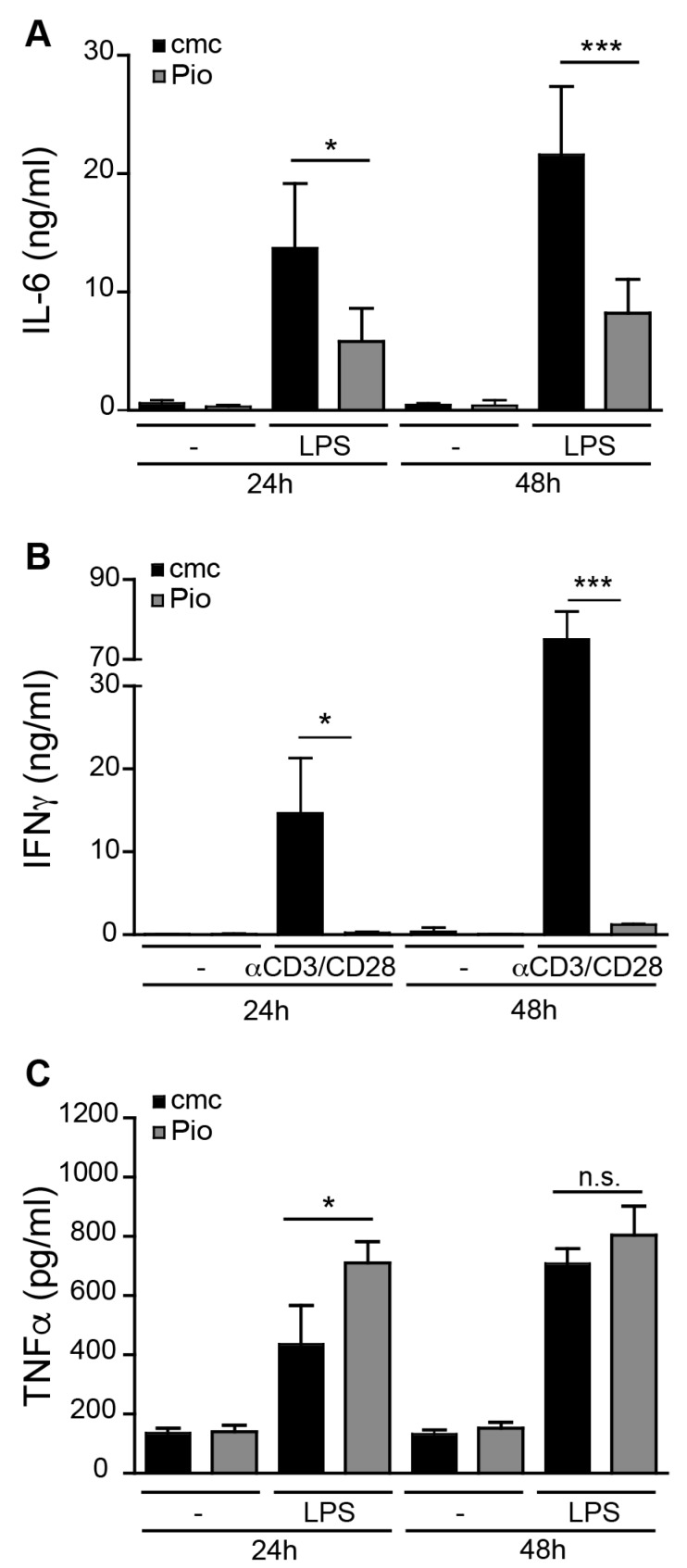
In vivo PPARγ activation reduces pro-inflammatory activity in LSEC and splenic T cells but not Kupffer cells ex vivo. C57BL/6 mice were fed for 7 days with 30 mg/kg Pioglitazone (Pio) or with vehicle (cmc) alone. (**A**) LSEC were isolated from the livers of these mice and stimulated in vitro with 100 ng/mL of the TLR4 ligand LPS or were left untreated. After 24 and 48 h, IL-6 content in the supernatant was determined by ELISA. (**B**) CD8 T cells were isolated from the spleen and stimulated with plate-bound anti-CD3ε/CD28 antibodies. Supernatants were analyzed for the IFNγ content by ELISA after 24 and 48 h. (**C**) Kupffer cells were isolated from Pio-treated and non-treated mice and incubated with 100 ng/mL LPS. TNFα content in the supernatant was measured after 24 and 48 h by ELISA. Data are presented as mean ± Standard Deviation (SD); one representative experiment out of three is shown. * *p* ≤ 0.05, *** *p* ≤ 0.001, n.s. = not significant.

In murine models of immune-mediated hepatitis, immune cells involved in the induction of liver damage include LSEC, CD4 T cells and Kupffer cells [13,14,15]. Thus, we investigated the effect of in vivo PPARγ activation on these immune cell populations. Mice were fed with the PPARγ ligand Pioglitazone (Pio) or the vehicle carboxy-methyl-cellulose (cmc) alone for 7 days, after which LSEC or Kupffer cells were isolated from the liver, or T lymphocytes from the spleen. As LSEC express TLR4 [16], we stimulated LSEC isolated from Pio- and vehicle-fed mice with the TLR4 ligand LPS and analyzed their production of the pro-inflammatory cytokine IL-6. LPS induced substantial IL-6 production by LSEC, which was significantly reduced when PPARγ was activated (Figure 1A). Similarly, PPARγ activation in vivo inhibited the function of splenic T cells as their ability to produce IFNγ (Figure 1B) ex vivo upon anti-CD3ε/CD28 stimulation was severely reduced over time (Figure 1B). Although the anti-inflammatory effects of PPARγ in macrophages have been well documented [2], in vivo PPARγ activation did not inhibit TNFα production by Kupffer cells ex vivo after LPS stimulation (Figure 1C), but increased early (24 h) but not late (48 h) TNFα production. Taken together, our data suggest that in vivo PPARγ ligation has potent anti-inflammatory effects on T cells and LSEC, but not on TNFα production by Kupffer cells.

### 2.2. PPARγ Activation in LSEC Does Not Influence T Cell Modulatory Capacity

**Figure 2:**
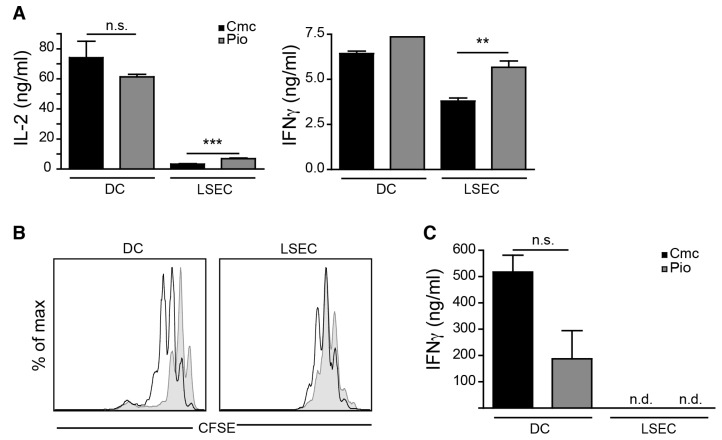
PPARγ activation does not alter T cell function after priming by LSEC. C57BL/6 mice were fed for 7 days with 30 mg/kg Pioglitazone or with vehicle (cmc) alone. LSEC and dendritic cells (DC) were isolated from these mice and cocultured with purified ovalbumin (OVA)-specific CFSE-labeled OT-1 CD8 T cells in the presence of 100 μg/mL OVA. (**A**) After 24 h, IL-2 and IFNγ release was determined by ELISA. (**B**) After 72 h, the proliferation profile of the OT-1 CD8 T cells was determined by flow cytometry (black line, no fill = cmc, grey line, grey fill = Pio). (**C**) After 4 days of coculture, CD8 T cells were re-stimulated with plate-bound anti-CD3ε/CD28 antibodies and 24 h later, production of IFNγ was measured by ELISA. Error bars indicate mean ± SD, *n* = 3; one representative experiment out of three is shown. ** *p* ≤ 0.01, *** *p* ≤ 0.001, n.s. = not significant, n.d. = not detected.

Although LSEC can produce pro-inflammatory cytokines, like IL-6, under homeostasis, LSEC display immune modulatory functions towards T lymphocytes [17]. Cross-presentation of systemic antigens by LSEC results in the induction of CD8 T cells lacking an immediate effector function, but possessing a memory function [18]. As we found that PPARγ activation influences LSEC function with respect to LPS-induced IL-6 production (Figure 1A), and as we have previously shown that T cell stimulatory function in dendritic cells is severely affected after PPARγ ligation [6,19], we analyzed the ability of PPARγ-activated LSEC to fulfill their function as regulatory antigen-presenting cells. First, we examined initial CD8 T cell stimulation by LSEC and compared it to splenic DC isolated from Pio- or vehicle-fed mice. Early IL-2 and IFNγ production by CD8 T cells was not affected by PPARγ ligation in antigen-presenting DC (Figure 2A). In contrast, IL-2 and IFNγ production in CD8 T cells activated by PPARγ-activated LSEC was increased after 24 h (Figure 2A). This difference was not reflected in the induction of proliferation in CD8 T cells, as both PPARγ-activated DC and LSEC induced a delay in T cell proliferation (Figure 2B). However, after re-stimulation of LSEC-primed or DC-primed CD8 T cells, PPARγ activation in DC reduced IFNγ production in DC-primed CD8 T cells, similar to what we found previously for CD4 T cell priming [6], but LSEC-primed T cells failed to produce IFNγ upon re-stimulation, irrespective of PPARγ activation (Figure 2C). In summary, these data show that although PPARγ activation in LSEC affects pro-inflammatory cytokine production, it does not alter their function with respect to CD8 T cell priming.

### 2.3. Pioglitazone-Mediated PPARγ Activation Aggravates GalN/LPS-Induced Hepatitis

**Figure 3:**
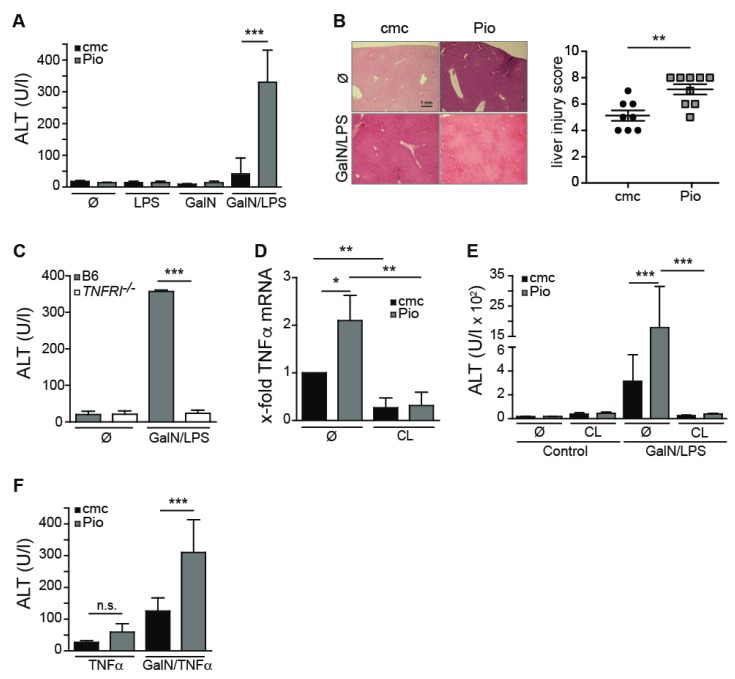
Oral administration of Pioglitazone sensitizes mice for immune-mediated experimental hepatitis. (**A**,**B**,**D**,**E**,**F**) C57BL/6 and (**C**) TNF receptor I (TNFRI)^−/−^ mice were fed with 30 mg/kg Pioglitazone or with vehicle (cmc) for 7 days. (**A**) Plasma ALT levels from C57BL/6 mice after 6–7 h after the i.p. injection of GalN, LPS, GalN/LPS or phospate buffered saline (PBS). (**B**) Exemplary H&E staining of livers of as-indicated at 20× magnification. The bar graph depicts a histological liver injury score as detailed in the Materials and Method Section of vehicle (*n* = 7) and vehicle + Pioglitazone (*n* = 9)-treated mice. (**C**) Plasma ALT levels 6–7 h after i.p. GalN/LPS or PBS injection into C57BL/6 and TNFRI^−/−^ mice that were fed with Pio for 7 days. (**D**) C57BL/6 mice that were fed with Pio or vehicle (cmc) for 7 days were injected with clodronate liposomes (CL) or were left untreated. Then, mice received GalN/LPS i.p. and 1 h later, intrahepatic TNFα mRNA levels were determined by real-time polymerase chain reaction (PCR). (**E**) Plasma ALT levels were determined 6–7 h after injection of GalN/LPS in clodronate liposome (CL)-injected and non-injected C57BL/6 mice. (**F**) Pio- and vehicle-fed C57BL/6 mice were injected with 400 ng TNFα 30 min post GalN injection or received GalN alone. Plasma ALT levels were measured 4 h later. Error bars indicate mean ± SD, *n* ≥ 4; one representative experiment (**A**, **C**–**F**) out of three is shown. * *p* ≤ 0.05, ** *p* ≤ 0.01, *** *p* ≤ 0.001, n.s. = not significant.

We next investigated if the anti-inflammatory effects of PPARγ acting on the various cell populations in the liver, that we observed in vitro, were sufficient to influence the course of liver inflammation in the GalN/LPS model [20] of immune-mediated hepatitis. To this end, we injected Pioglitazone- or vehicle-fed mice with GalN/LPS and measured plasma alanine aminotransferase (ALT) levels 6–7 h later. Unexpectedly, the administration of GalN/LPS to Pio-fed mice did not ameliorate but instead significantly exacerbated liver injury, as measured by plasma ALT levels (Figure 3A) and a significantly higher liver injury score in histology (Figure 3B). As we found that Kupffer cells from Pioglitazone-fed mice increased their early TNFα secretion in vitro (Figure 1C), we next investigated whether the observed increased liver injury after Pioglitazone administration in GalN/LPS administration in Pio-fed mice involves TNFα.

We first established that after GalN/LPS injection, the production of TNFα was essential for the ensuing liver injury, as GalN/LPS injection in TNFRI-deficient mice, unable to respond to TNFα, did not induce any liver injury, as measured by plasma ALT levels (Figure 3C). We could further show that in Pio-fed mice that were challenged with GalN/LPS, intrahepatic TNFα mRNA levels were increased (Figure 3D). Macrophages/Kupffer cells are the main producers of TNFα in the GalN/LPS model [21], and the activation of PPARγ by Pioglitazone in macrophages has been described to diminish TNFα production by these cells [1]. However, our results demonstrated that in vivo Pioglitazone administration does not reduce LPS-induced TNFα production by Kupffer cells (Figure 1C). To investigate whether Kupffer cells were responsible for the increased intrahepatic TNFα mRNA levels after GalN/LPS injection in Pio-fed animals, we depleted Kupffer cells by the administration of clodronate liposomes (CL) to Pio- or vehicle-fed mice before administration of GalN/LPS (Figure 3D). In the absence of Kupffer cells, we did not detect elevated TNFα mRNA levels in Pio-fed compared to control-fed mice (Figure 3D). Moreover, in CL-treated animals, we did not observe any increase in TNFα mRNA levels (Figure 3D), nor did these mice develop hepatitis with increased plasma ALT levels (Figure 3E), indicating that Kupffer cells producing increased levels of TNFα were the primary cause of augmented liver damage after GalN/LPS in Pio-fed mice. Interestingly, we could previously show in a model of hepatic adenovirus infection, that hepatocytes can develop an increased sensitivity towards TNFα-mediated signaling after infection [22]. To test whether hepatocytes show such heightened sensitivity, we administered a set concentration of recombinant TNFα, instead of LPS, to GalN-treated mice. When receiving the same amount of TNFα in combination with GalN, the Pio-fed mice had higher plasma ALT levels (Figure 3F), indicating that Pioglitazone administration not only induces higher levels of TNFα but at the same time, increases the sensitivity of GalN-treated hepatocytes towards the produced TNFα. Interestingly, even in the absence of GalN administration, the injection of TNFα resulted in a slight increase in plasma ALT in Pio-fed mice compared to vehicle-fed mice, suggesting that Pioglitazone by itself is capable to sensitize hepatocyte towards the death-inducing effect of TNFα. Taken together, our data suggest that the administration of Pioglitazone worsens the outcome of immune-mediated hepatitis by a combination of unexpected induction of higher levels of TNFα in Kupffer cells and an additional sensitization of hepatocytes towards TNFα-induced death.

## 3. Discussion

Activation of the anti-inflammatory transcription factor PPARγ is known to reduce production of inflammatory mediators in macrophages and dendritic cells [6,23]. Furthermore, PPARγ activation has been described to downregulate inflammatory responses [10,24] and can either directly [9] or indirectly affect T cell immunity [6]. Also, in our experiments, PPARγ activation had strong immunomodulatory effects in T cells and almost completely inhibited effector cytokine production following activation through the T cell receptor. We additionally found that PPARγ activation in liver-resident LSEC reduced the production of pro-inflammatory cytokines like IL-6 after LPS stimulation but did not significantly influence their function with regards to CD8 T cell priming. Although PPARγ activation in macrophages has been reported to result in marked inhibition of TNFα production [1], it did not decrease in the case of liver-resident Kupffer cells, but temporarily increased TNFα production upon TLR stimulation after PPARγ activation. Together, these results demonstrate that the consequences of pharmacologic PPARγ activation vary between different immune cells or even between macrophages from different organs, and that liver macrophages are non-responsive towards the anti-inflammatory effects of pharmacologic PPARγ activation.

To study the in vivo effects of PPARγ activation in immune-mediated hepatitis, in which the interplay between immune and non-immune cells is pivotal for liver injury, we analyzed the effect of pharmacologic PPARγ activation in the experimental hepatitis model based on the application of Galactosamine and LPS. Most strikingly, we found that PPARγ activation by Pioglitazone administration did not reduce but rather exacerbated GalN/LPS hepatitis. These findings in GalN/LPS hepatitis were unexpected as it has been reported that increased hepatic PPARγ expression during GalN/LPS hepatitis was associated with decreased severity [12], and we observed that isolated immune cell populations, such as LSEC and T cells, responded to pharmacologic PPARγ activation with reduced pro-inflammatory cytokine production. Although dendritic cells and macrophages have been shown to secrete less inflammatory cytokines after Pioglitazone-mediated PPARγ activation [2,6], our experiments indicate that the effect of Pioglitazone administration on single isolated hepatic immune cell populations cannot predict the influence of pharmacologic PPARγ activation in vivo in an experimental model of hepatitis. Critical to the pathogenesis of liver damage in GalN/LPS-induced hepatitis is the expression of TNFα. As we could show that following application of Pioglitazone, TNFα gene expression is increased in GalN/LPS-treated mice, it is possible that this leads to the observed increase in ALT levels in the plasma of these mice. The elevated TNFα levels upon GalN/LPS injection in Pioglitazone-treated animals may be attributed to the increased production of TNFα by hepatic macrophages. Indeed, ex vivo TLR-stimulated Kupffer cells from Pioglitazone-fed mice showed a tendency to produce more TNFα in vitro. Under non-inflammatory conditions, the liver is constantly exposed to low levels of LPS coming from the gastrointestinal tract, leading to a reduced sensitivity of hepatic antigen-presenting cells to subsequent LPS-mediated stimulation [25,26], which is called LPS/endotoxin tolerance [27]. The induction of such LPS tolerance is dependent on TLR4 signaling resulting in an initial NF-κB activation [27], after which a reduced sensitivity to further pro-inflammatory stimuli is established. The activation of PPARγ by Pioglitazone, which inhibits NF-κB-mediated transcription [2], may prevent the induction of such endotoxin tolerance by inhibiting the initial TLR-dependent NF-κB activation and thus resulting in an increased TNFα production by macrophages in Pioglitazone-fed mice that are challenged with LPS. Exacerbating the effects of overproduction of TNFα by liver macrophages in this system could be the reciprocal decrease in anti-inflammatory IL-10 production by macrophages [28], that has been reported to be able to significantly reduce or even prevent GalN/LPS-induced liver injury [29]. Conversely, inhibiting PPARγ via antagonist administration could reverse this situation, possibly leading to a protective effect in GalN/LPS-induced liver injury.

The family of thiazolidinediones is widely used to activate PPARγ in the treatment of type II diabetes. Interestingly, Troglitazone, like Pioglitazone, a member of the thiazolidinediones, can cause PPARγ-independent hepatotoxicity, potentially leading to hepatic organ failure [30,31]. It is thought that during metabolization of Troglitazone in the liver, chemically reactive metabolites may be formed, potentially leading to hepatocyte apoptosis and hepatotoxicity [32,33]. Although Pioglitazone administration is not associated with lethal liver injury in patients, Pioglitazone administration, similar to Troglitazone, leads to elevated plasma ALT levels in patients [34], indicating that Pioglitazone may cause minimal damage to the liver. Our results indicate that Pioglitazone administration leads to an increase in liver damage after GalN/LPS administration due to elevated TNFα production by liver-resident macrophages. Additionally, our data suggest that Pioglitazone increases the susceptibility of hepatocytes to the death-inducing effects of TNFα. Indeed, some case reports suggest that in human patients, receiving Pioglitazone as a treatment, for instance, for Type II diabetes, a similar course of events may be possible, leading to acute liver damage with high ALT, which is reversed as soon as Pioglitazone treatment is discontinued [35,36]. However, such increased sensitivity of hepatocytes towards TNFα may not be restricted to drug-induced stress in hepatocytes, but may also occur when hepatocytes are virally infected, which also leads to higher liver damage upon injection of TNFα [22].

Although the activation of PPARγ by synthetic ligands has been widely accepted to have anti-inflammatory effects and even provide protection in experimental models of inflammation in the central nervous system (CNS) and the bowel, we provided evidence here that in the liver, the pharmacological activation of PPARγ by Pioglitazone does not protect from immune-mediated damage but instead has detrimental effects on the disease course. While our observations indicate that Pioglitazone exacerbates immune-mediated injury and thereby provides an interesting in vivo model for further research on drug-induced liver injury, the molecular and cellular mechanisms causing this augmented liver injury will require further investigation.

## 4. Materials and Methods

### 4.1. Mice

C57BL/6, H2-K^b-SIINFEKL^-restricted OT-I T Cell Receptor-transgenic and TNFRI^−/−^ mice were bred in the central animal facility in Bonn under specific pathogen-free conditions according to the Federation of European Laboratory Animal Science Association (FELASA) and were used for experiments at 6 to 10 weeks of age. The protocols for animal experiments were approved by the local government of North-Rhine Westphalia and the Behörde für Gesundheit und Verbraucherschutz of the Freie und Hansestadt Hamburg (permit number 8.87-50.10.37.09.137 and N002/18). All efforts were made to minimize suffering.

### 4.2. Reagents

Pioglitazone (Actos; Takeda Pharmaceuticals, Berlin, Germany) treatment of mice was performed by daily oral gavage of 30 mg/kg body weight suspended in 0.5% carboxymethylcellulose (cmc, Sigma-Aldrich, Munich, Germany), or vehicle only 7 days before cell isolation or induction of experimental hepatitis in vivo. For in vitro cell culture, 10 µM Pioglitazone (Pio) was added into the medium daily. LSEC and Kupffer cells were stimulated with 100 ng/mL LPS (Sigma-Aldrich, Munich, Germany).

### 4.3. Cell Isolation

LSEC were isolated as described previously [36] and purified by immunogenic separation using αCD146-microbeads (Miltenyi Biotec, Bergisch Gladbach, Germany), achieving purity of >98% and plated into collagen-coated 24-well or 96-well plates. One day after isolation, cells were washed with PBS containing 1% Fetal Calf Serum (FCS) and used two days later for experiments. Kupffer cells were isolated after perfusion of the liver with a collagenase solution and subsequent digestion with collagenase in vitro. After a 50%/25% Percoll gradient (GE Health Care, Freiburg, Germany), cells were plated, incubated at 37 °C and washed after 30 min. Adhering Kupffer cells were then used for experiments. After LPS stimulation of LSEC and Kupffer cells, supernatants were harvested at 24 and 48 h and IL-6 content was measured by ELISA. Dendritic cells (DC) were purified by immunogenic separation using αCD11c-microbeads (Miltenyi Biotec, Bergisch Gladbach, Germany) after collagenase-digestion of spleens according to the manufacturer’s instructions. CD8+ T cells were isolated from spleens and lymph nodes of H2-Kb-SIINFEKL-restricted OT-I TCR-transgenic mice by αCD8-microbeads (Miltenyi Biotec).

### 4.4. T Cell Assays

CD8 T cells were stimulated with plate-bound anti-CD3ε/CD28 antibodies (anti-CD3ε; clone 145.C11, anti-CD28; clone 37.51) in a 96-well plate. After 24 and 48 h, supernatants were analyzed for IFNγ content by ELISA. Carboxyfluorescein Succinimidyl Ester (CFSE)-labeled naïve OT-1 CD8+ T cells were cocultured with LSEC or DC from Pio- and vehicle-fed mice in the presence of 100 μg/mL OVA. After 24 h, the IFNγ and IL-2 content was determined in the supernatant via ELISA. Additionally, after 72 h, T cell proliferation was determined by flow cytometry. Acquisition and analysis were conducted using a FACS Canto II (BD, Heidelberg, Germany) and FlowJo software (TreeStar Inc., Ashland, OR, USA). After 4 days of coculture, viable CD8 T cells were isolated via density gradient centrifugation (Lonza, Cologne, Germany), re-stimulated on anti-CD3ε-coated plates and supernatants were analyzed for IFNγ content by ELISA 24 h later.

### 4.5. Experimental Hepatitis (GalN/LPS, GalN/TNFα)

All reagents were dissolved in pyrogen-free saline. GalN/LPS-mediated liver injury was induced by simultaneous i.p. injection of 20 mg GalN (Sigma-Aldrich, Munich, Germany) and 10 ng LPS (Sigma-Aldrich, Munich, Germany). In the GalN/TNFα model, 400 ng murine recombinant TNFα (Invitrogen, Karlsruhe, Germany) was injected i.v. after i.p. GalN injection. Plasma ALT levels were measured using the scil Reflovet^®^Plus analyzer (Roche, Penzberg, Germany) at the indicated times. Liver tissue was isolated for histology, RNA isolation, cDNA synthesis and quantitative PCR at the indicated times. Quantification of intrahepatic cytokine mRNA levels was performed by qPCR as described previously [37].

### 4.6. Histology

Liver tissue was formalin-fixed and then processed for routine paraffin embedding. Standard protocols for Hematoxylin and Eosin (H&E) staining were used. A pathologist scored sections blindly. The liver injury score summarizes the score for plasma and lymphocytic infiltrate (0–3), granulocytic infiltrate (0-3), erythrocyte extravasation (0–3) and area of acute liver damage (0% = 0, 0%–30% = 1, 31%–60% = 2, 61%–100% = 3).

### 4.7. Depletion of Macrophages by Clodronate Liposomes

Mice were injected intravenously with liposome encapsulated dichloro- methylene- bisphosphonate (Cl_2_MBP) [38] 3 and 1 day before inducing hepatitis.

### 4.8. Statistical Analysis

Student’s *t* or analysis of variance (ANOVA) tests were used to determine statistical significance of the results. Data are depicted as the mean ± standard deviation (SD), and *p*-values < 0.05 were considered significant (* *p* < 0.05, ** *p* < 0.01, *** *p* < 0.001).
